# The z-spectrum from human blood at 7T

**DOI:** 10.1016/j.neuroimage.2017.10.053

**Published:** 2018-02-15

**Authors:** Simon M. Shah, Olivier E. Mougin, Andrew J. Carradus, Nicolas Geades, Richard Dury, William Morley, Penny A. Gowland

**Affiliations:** a Sir Peter Mansfield Imaging Centre, School of Physics and Astronomy, University of Nottingham, Nottingham, UK; b NIHR Nottingham Biomedical Research Centre, Nottingham University Hospitals NHS Trust, University of Nottingham, Nottingham, UK

**Keywords:** Chemical exchange saturation transfer (CEST), Nuclear overhauser enhancement (NOE), Z-spectrum, Magnetisation transfer, Blood relaxometry

## Abstract

Chemical Exchange Saturation Transfer (CEST) has been used to assess healthy and pathological tissue in both animals and humans. However, the CEST signal from blood has not been fully assessed. This paper presents the CEST and nuclear Overhauser enhancement (NOE) signals detected in human blood measured *via* z-spectrum analysis. We assessed the effects of blood oxygenation levels, haematocrit, cell structure and pH upon the z-spectrum in *ex vivo* human blood for different saturation powers at 7T. The data were analysed using Lorentzian difference (LD) model fitting and AREX (to compensate for changes in T_1_), which have been successfully used to study CEST effects *in vivo*. Full Bloch-McConnell fitting was also performed to provide an initial estimate of exchange rates and transverse relaxation rates of the various pools. CEST and NOE signals were observed at 3.5 ppm, −1.7 ppm and −3.5 ppm and were found to originate primarily from the red blood cells (RBCs), although the amide proton transfer (APT) CEST effect, and NOEs showed no dependence upon oxygenation levels. Upon lysing, the APT and NOE signals fell significantly. Different pH levels in blood resulted in changes in both the APT and NOE (at −3.5 ppm), which suggests that this NOE signal is in part an exchange relayed process. These results will be important for assessing *in vivo* z-spectra.

## Introduction

Z-spectrum features, in particular chemical exchange saturation transfer (CEST) have recently been used to study both healthy and pathological tissues *in vivo* (van Zijl and Yadav, 2011, Zaiss and Bachert, 2013, Liu et al., 2013), but so far there has only been one study of the z-spectrum of blood (Zheng et al., 2014). The z-spectrum provides a means of indirectly studying proton pools that are conventionally invisible to MRI, opening up the possibility of metabolic imaging with MRI (Cai et al., 2012, Walker-Samuel et al., 2013, Rivlin et al., 2013, van Zijl et al., 2007, Haris et al., 2014, Cai et al., 2015).

CEST (Zaiss and Bachert, 2013, Ward et al., 2000, Zhou and van Zijl, 2006) uses the principles of magnetisation transfer (MT) to investigate moieties containing labile protons by applying saturation at their resonant frequency and detecting the subsequent transfer of the saturation to the water pool *via* chemical exchange. The transferred saturation can only be detected if the chemical exchange is fast compared to the longitudinal recovery rate but in the slow to intermediate regime compared to the chemical shift between the water and the labile protons. Amide proton transfer (APT) CEST is associated with mobile amide protons at +3.5 ppm relative to water. It is sensitive to pH (Zhou et al., 2003) and has been applied to study ischemia and tumour grading, but its origins are not fully understood. The increase in APT in tumours has been attributed to an increase in the content of endogenous cellular proteins and peptides, despite the fact that the total content of proteins has been shown to be relatively constant in tumours (Yan et al., 2015). Amines (NH_2_) at 2.2 ppm are in faster exchange than APT, but are infrequently detected *in vivo* since high saturation powers are required. The amine signal has previously been used as a marker for pH (McVicar et al., 2014). The NOE signal at −3.5 ppm was believed to originate from regions of high myelination (Mougin et al., 2013, Jones et al., 2013) however, more recently *Xu* et al. showed that there is no NOE contrast difference between grey and white matter when the magnetisation transfer contrast is suppressed (Xu et al., 2016). The NOE signal may also be decreased in tumours (Zaiss et al., 2015) but this is probably related to increased water content and requires further investigation. *Jones* et al. also investigated multiple NOE peaks associated with the macromolecule MRS spectrum (Jones et al., 2013), and *Zhang* et al. observed a *‘new NOE peak around -1.6 ppm’* and showed it was reduced in ischemic stroke (Zhang et al., 2016).

*Zheng* et al. found that blood has increased CEST contrast relative to some other tissues (Zheng et al., 2014). However they assessed the CEST signal using MT asymmetry (Zhou et al., 2004), which is sensitive to competing effects of NOE (−3.5 ppm) and APT (+3.5 ppm) particularly at high field.

Asymmetry analysis was the original method used to analyse CEST and NOE signals from *in vivo* MRI but recently alternative quantification approaches have been proposed, such as Lorentzian difference (LD) fitting and AREX (Zaiss et al., 2011). LD fitting can characterise slow and intermediate exchanging proton pools but is not suitable for faster exchanging proton pools (such as amines and hydroxyls) which coalesce with the water signal (Zaiss and Bachert, 2013). To directly estimate the absolute sizes of the exchanging pools relative to water it is necessary to fit the data to a multi-pool Bloch-McConnell (BM) model (van Zijl and Yadav, 2011, Chappell et al., 2013, Geades et al., 2017). BM fitting is more time consuming than LD fitting and AREX, requires some *a priori* knowledge of parameters such as T_2_ and k_ex_ of each exchanging proton pool, and generally requires larger datasets to be used in the fit.

This work aims to investigate the CEST and NOE signals visible in *ex vivo* blood, considering the effects of oxygenation, haematocrit, cell structure and pH, quantifying the results using AREX and also direct BM fitting to provide initial estimates of the exchange rates and transverse relaxation times of the various pools.

## Methods

### *In vivo* human data

With local ethical approval, 2 male subjects aged 31 and 35 were recruited and z-spectra were acquired from the head.

### Blood collection and preparation

With local ethical approval, a total of 98 ml of venous blood was taken from 4 healthy male volunteers and was stored in vials containing an anticoagulant (heparin) or allowed to clot. The unclotted samples were then treated in various ways with the number of samples used for each treatment shown in Table 1.

**Table 1:** The number of blood samples taken during the experiments across the 4 healthy male volunteers.

Type of sample	Number of Samples
Control (4 ml)	4
Oxygenated (4 ml)	4
Deoxygenated (4 ml)	3
Concentrated RBCs (2 × 4ml)	4
Plasma (2 × 4ml)	2
Clotted (5 ml)	2
pH 6.8 (4 ml)	1
pH 7.0 (4 ml)	1
pH 7.4 (4 ml)	1
**Total**	24

Either 120 ml of nitrogen gas or medical (100%) oxygen was bubbled through the blood to deoxygenate or oxygenate it. To achieve this two syringe needles were pushed through the rubber seal of the vials, one connected to a 60 ml syringe to the relevant gas, and the second to exhaust gas from the vial to prevent pressure rise. A very low gas flow rate was used to prevent foaming (Gardener et al., 2010) and the vials were regularly inverted to ensure good mixing. A blood gas analyzer (i-STAT, Abbott) was used to measure the samples’ saturation of oxygen (SO_2_), partial pressure of oxygen (PO_2_), pH and haematocrit (Hct) levels.

8 ml of unmodulated blood was spun in a centrifuge (15 min at 800 rpm) and the plasma was removed. Two such samples were combined to give a concentrated RBC (cRBC) sample with 40% greater Hct level than whole blood. The remaining 12 ml of blood samples were washed with a phosphate saline buffer to modify the pH to 6.8, 7.0 and 7.4. MR scanning was commenced within one hour of blood collection. At the end of scanning, the blood was frozen and defrosted within a week to lyse the blood cells before repeating the scan. This method of lysis causes cells in the blood to swell and ultimately break as ice crystals form during the freezing process. The cell skeletons were left in the samples during the repeated scanning.

A sample of heparinized water was also scanned.

For MR scanning the vials were placed in a temperature controlled holder. The holder was designed to allow warm water to flow slowly around the samples to keep them at 37 °C (PMT Instruments). The holder was rotated every 4 minutes during scanning to limit blood sedimentation, and could be repositioned after each rotation with a mean displacement of 0.01 ± 0.02 mm in the x-direction and 0.11 ± 0.05 mm in y-direction.

### MRI acquisition

**Fig. 1:**
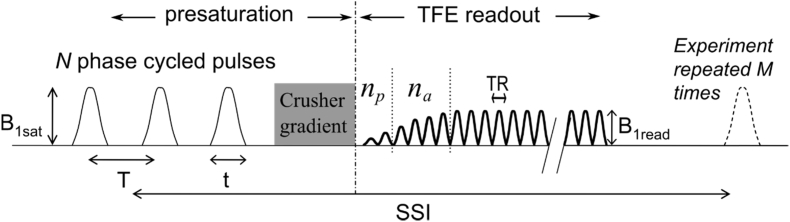
The magnetisation transfer-prepared turbo field echo (MT-TFE) sequence: a presaturation period followed by the TFE readout. The presaturation period consists of N = 40 Gaussian windowed sinc pulse. The duty cycle of 50% was used such that t = 30 ms and T = 60 ms. The crusher gradient at the end of the saturation train removes any residual transverse magnetisation. n_p_ = 2 and n_a_ = 4 are the number of ramped RF pulses before and at the start of the acquisition, respectively. From (Mougin et al., 2013).

All MRI experiments were conducted on a 7T Philips Achieva with a volume transmit RF coil and Nova 32 channel receive coil. Z-spectra were obtained using a saturation-prepared 3D TFE sequence (Mougin et al., 2013) shown in Fig. 1. The pulse sequence involved a train of 40 Gaussian windowed sinc RF pulses with bandwidth 200 Hz and a duty cycle of 50% applied prior to a TFE readout with a low-high radial k-space acquisition scheme.

The pulses were phase cycled and a spoiler gradient was applied at the end of the pulse train to remove residual transverse magnetisation. Further details are given in Table 2.

**Table 2:** MR sequence parameters for the blood samples and *in vivo* acquisition.

	Blood samples	*In vivo*
B_1max_ (μT)	2.4, 4.9 and 8.5	2.4 and 6.0
B_1,rms_ (μT)	0.43, 0.87 and 1.52	0.43 and 1.1
Off resonance frequencies (ppm)	83 off-resonance frequencies between ± 17 ppm, and at 50 kHz (167 ppm).	38 and 23 off-resonance frequencies between ±33 ppm, and at 50 kHz (167 ppm) for 2.4 μT and 6.0 μT respectively
FOV (mm^3^)	224 × 224 x 48	192 × 186 x 50
Resolution (mm^3^)	1.5 × 1.5 x 2	1.5 × 1.5 x 1.5
TE/TR/FA	2.6 ms/5.55 ms/8°	2.8 ms/6 ms/8°
SENSE (RL)	2	2
Number of TFE shots	5	4

An additional acquisition was made with a presaturation at 167 ppm, which was assumed not to saturate any exchangeable magnetisation. This data point was used to estimate M_0_ for normalisation, since we had previously observed that off-resonance pre-saturation resulted in a small change in the amplitude of the readout pulses.

B_0_ and B_1_ (AFI (Yarnykh, 2007)) maps were acquired to correct for the field inhomogeneities (allowing no change in transmitter calibration between acquisition of the B_1_ map and the z-spectrum). T_1_ maps were acquired using an inversion recovery sequence with FOV 224 × 224 × 48 mm^3^ and voxel size 1.5 × 1.5 × 2 mm^3^, TI = 150, 200, 300, 400, 500, 600, 700, 800, 1000, 1200, 1500, 1800, 2200, 3000, 3500, 4000, 4500 and 5000 ms, TR/TE = 6.9/3.2 ms and SENSE factor (RL) of 2. T_2_* maps were acquired using a multi-gradient echo (echos = 8) with FOV 256 × 256 × 48 mm^3^ and voxel size 2 × 2 × 2 mm^3^. TR/TE = 45/1.35 ms and ΔTE = 1.8 ms.

### Data processing

Voxel-wise z-spectra were obtained for each RF saturation power by calculating

(1) $$\;Z\left(\text{Δ}\omega \right)=\;M_{z,sat}\left(\text{Δ}\omega \right)/M_{0}$$

at each off-resonance (Δω), with normalisation by the signal M_0_ acquired at 50 kHz (167 ppm). The z-spectra were B_0_ corrected by interpolating to 0.01 ppm resolution between ±17 ppm, then shifting the entire spectrum by the difference between the B_0_ map and the zero point at 0 ppm, and finally re-sampling back to the original off-resonance frequencies (Zaiss and Bachert, 2013, Zhou et al., 2003).

The B_1_ map was used to estimate the actual saturation amplitude at which each z-spectrum from each voxel was acquired. Next the z-spectra for the target B_1_ amplitudes of 0.5, 0.9 and 1.3 μT were estimated by using spline interpolation between measured z-spectra up to 1.5 μT, including an additional point at 0 μT (Windschuh et al., 2015).

The T_1_ relaxation time was calculated by fitting the inversion recovery data to $M=M_{0}(1-\alpha \;\text{exp}\left(-TI/T_{1}\right)$ where α accounted for incomplete inversion. T_2_* was calculated by fitting the natural log of 8 gradient echoes to a linear decay using a weighted least-squared fitting algorithm, excluding any data that fell below background noise.

To quantify changes in CEST and NOE components, initially Lorentzian difference (LD) model fitting was performed (Zaiss et al., 2011), assuming that the z-spectra could be approximated by a linear combination of Lorentzian functions corresponding to amides (nominally at 3.5 ppm), amines (nominally at 2.2 ppm), the NOEs (nominally at −3.5 and −1.7 ppm), and a Lorentzian corresponding to the MT from macromolecules (Hua et al., 2007, Morrison and Henkelman, 1995). The LD model was fitted to the B_1_ corrected z-spectra using a least-square algorithm, allowing the positions of the CEST and NOE peaks to vary by ± 0.4 ppm around the predicted literature values, and the amplitude and width of the peaks to vary. The variations in the position and width of the peaks accounted for the differences in T_2_* and frequency offset between oxygenated and deoxygenated blood.

To account for changes in T_1_, the AREX signal (Zaiss et al., 2015) was calculated,

(2) $$AREX(\text{Δ}\omega )=\left(\frac{1}{Z_{lab}(\text{Δ}\omega )}-\frac{1}{Z_{ref}(\text{Δ}\omega )}\right)/T_{1}\text{,}$$

where T_1_ was the observed longitudinal relaxation time, Z_lab_(Δω) was the measured Z-value (Equ. 1) at off-resonance frequency Δω and Z_ref_(Δω) was the Z-value from the summation of all fitted Lorentzians, except for the Lorentzian for the proton pool of interest at Δω. Whilst AREX can compensate for changes in T_1_ between the fresh and lysed blood samples, it cannot be computed over the total width of the Lorentzian lineshape, and therefore the peak amplitude of the fitted Lorentzian was used. AREX assumes steady state has been achieved and breaks down close to 0 ppm due to the inverse in Equ (2), and therefore it is difficult and sometimes impossible to calculate AREX values for the amines (2.2 ppm) and NOE_-1.7ppm_ (Heo et al., 2017). Additionally the amine (and other hydroxyls pool) peaks coalesce with the water peak, making them difficult to separate the signals when performing Lorentzian lineshape fitting.

We also fitted the z-spectra acquired at three B_1_ saturation powers (162 data points in total) jointly to a 6-pool BM model (Woessner et al., 2005) to provide absolute estimates of the pool sizes M_i,0_/M_f,0_, T_i2_, and exchange rate k_i,ex_ of each (*i*th) exchanging proton pool in an iterative manner. These fits were carried out on the University of Nottingham High Performance Computer. The model only allowed for exchange between the off-resonance pools and free water, assuming that direct exchange between the other pools was negligible. Initially the three spectra from the control blood sample were fitted to the 6-pool model using a genetic algorithm to obtain an initial estimate of the 16 parameters (5 M_i,0_/M_f,0,_ 6 T_i2_, and 5 k_i,ex_), assuming the water longitudinal relaxation time measured with the inversion recovery experiment, and the RF amplitude from the B_1_ map. The starting values for this fit are given in Table 3; these values were taken from brain data (van Zijl et al., 2017) adjusted from preliminary BM fits. We made an estimate of the errors in the fitted parameters by considering the span of possible fitted parameter values consistent with doubling in the sum of squares at the fitted value (which would correspond to halving the signal to noise ratio (SNR) in the spectra if the error in the fit were purely due to random noise).

**Table 3:** Starting parameters used for the 6 pool Bloch-McConnell fitting of the z-spectra acquired on the control blood sample for B_1_ saturation powers of 0.43, 0.87 and 1.52 μT.

	APT	Amines	NOE_-1.7ppm_	NOE _-3.5ppm_	MT
T_2_ (ms)	0.5	10	1.2	0.5	0.01
Exchange rate (Hz)	20	500	1.5	5	15
Assumed frequency offset from water (ppm)	3.5	2.2	−1.7	−3.5	−2.34
Assumed T_1_ (ms)	1000	1000	1000	1000	1000

Next these results were used to estimate the pool sizes M_i,0_/M_f,0_ for the fresh and lysed control and concentrated samples, assuming no change in T_i2_, and k_i,ex_. This approach allowed better comparison of the relative pool sizes than if T_i2_, and k_i,ex_ had been allowed to vary freely. The data from each sample were fitted for 5 parameter to the 6-pool model. For each sample the T_2_ of water was estimated from the linewidth of the direct saturation (DS) peak within 400 Hz of resonance.

## Results

**Fig. 2:**
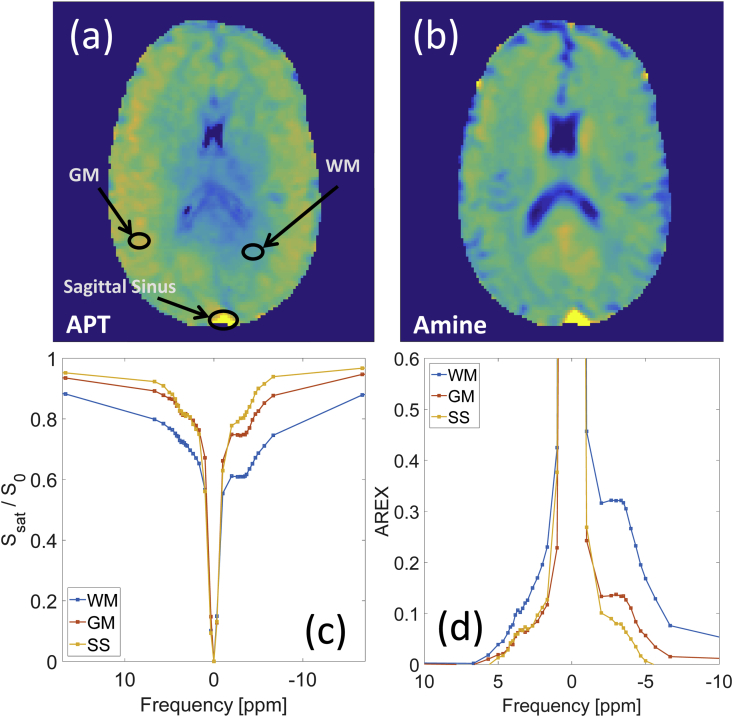
Lorentzian difference maps (B_1_ corrected to a target of 0.5 μT) for (a) APT and (b) amine maps. The corresponding (c) z-spectra (d) AREX spectra (with the MT contribution removed) for the three ROIs indicated on the APT map are also shown.

*In vivo* APT and amine maps from one volunteer produced by LD fitting are shown in Fig. 2(a–b). Fig. 2(c–d) show the z-spectra and AREX spectra for the regions of interest indicated in Fig. 2(a). Both spectra suggest high APT and amine signals and low NOE signals in the sagittal sinus compared to white matter and grey matter. Similar results were obtained in the other volunteer. Fig. 2(c) shows that the MT asymmetry observed in WM and GM is not present in blood.

**Fig. 3:**
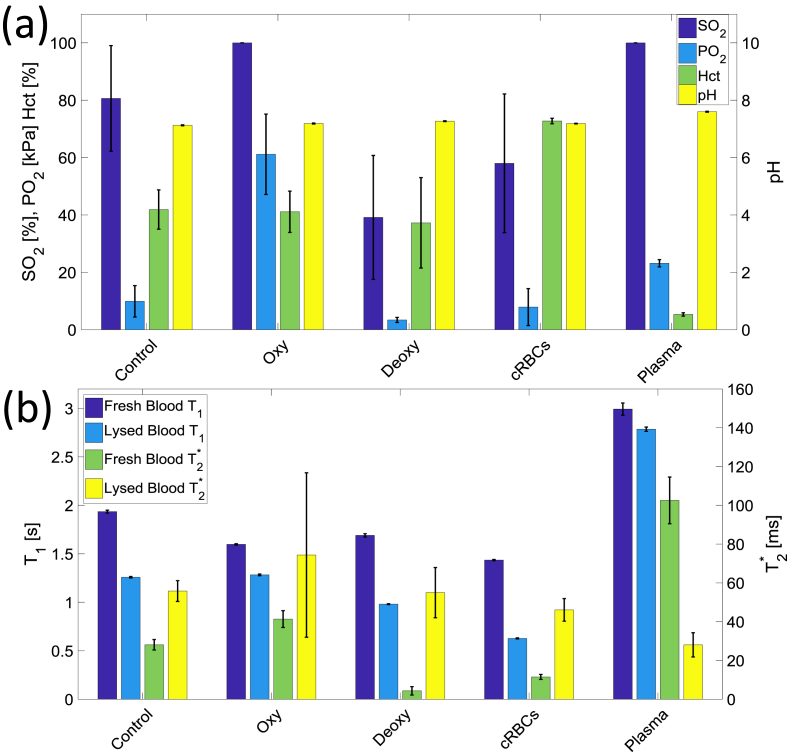
(a) The saturation of oxygen (SO_2_), partial pressure of oxygen (PO_2_), pH levels and the haematocrit levels of the control, oxygenated, deoxygenated, concentrated RBCs and plasma blood samples, and (b) the average T_1_ and T_2_* for fresh and lysed blood samples separately.

Fig. 3 shows the (a) oxygen saturation (SO_2_), partial pressure of oxygen (PO_2_), haematocrit, pH and (b) average T_1_ and T_2_*, of each set of samples. For all oxygenated samples the SO_2_ reached 100% after preparation and maintained this level for the duration of the experiment. The SO_2_ of the deoxygenated and control blood was approximately 40% and 80% respectively. Both samples’ oxygenation levels changed during the experiment (control SO_2_ increased and deoxygenated SO_2_ decreased) because the gas in the space above the blood in the vials continued to exchange with the red blood cells. The oxygenated and deoxygenated blood had a PO_2_ of approximately 60 and 3 mmHg respectively. The pH levels for all the samples were between 7.3 and 7.5 during scanning, with a drop of pH 0.2–0.3 detected over the course of scanning. The Hct was approximately 40% for the control, oxygenated and deoxygenated blood, over 70% for the concentrated RBCs and less than 10% for plasma.

Fig. 3(b) shows that the T_2_* of the deoxygenated blood was approximately five times shorter than that of the oxygenated and control blood samples. The concentrated RBCs had a shorter T_2_* than the oxygenated and control blood (and also a somewhat lower SO_2_), and the plasma had a much longer T_2_* than all the other samples. Lysing the blood shortened the T_1_ and lengthened T_2_* for all the blood samples.

The z-spectrum of heparinized water only showed the DS peak.

**Fig. 4:**
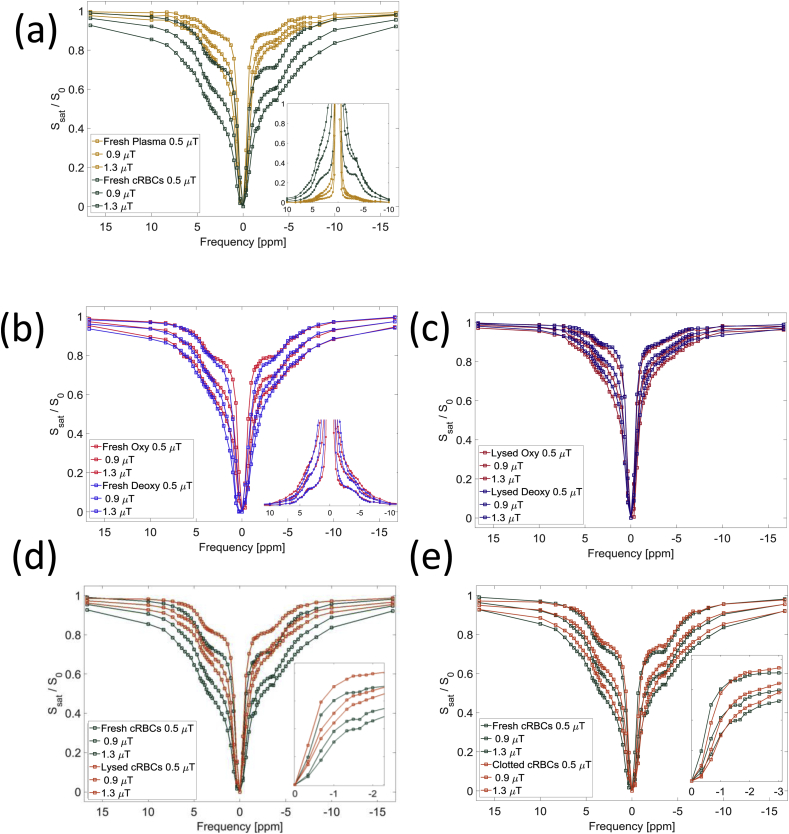
B_1_ corrected z-spectra for the (a) fresh plasma and concentrated RBCs with (inset) the AREX spectra for the fresh cRBCs and plasma, (b) fresh oxygenated and deoxygenated blood with (inset) the AREX spectra for the fresh oxygenated and deoxygenated blood, (c) B_1_ corrected z-spectra for the lysed oxygenated and deoxygenated blood with (inset) a zoom in around −1.7 ppm, (d) fresh and lysed concentrated RBCs and (e) fresh and clotted concentrated RBCs with (inset) a zoom in around −1.7 ppm. All are presented at the target B_1_ saturation powers of 0.5, 0.9 and 1.3 μT.

Figure 4(a) shows B_1_ corrected example z-spectra for concentrated RBCs and plasma, including an inset of the AREX spectra. The longer T_2_* of plasma gave a narrower lineshape around 0 ppm, but these spectra indicate that the largest source of the MT, CEST and NOE signal in the blood is from the RBCs rather than plasma. Fig. 4(b) shows no significant difference in NOE and CEST peaks between the z-spectra for oxygenated and deoxygenated blood. The wider DS peak of the deoxygenated blood is expected due to its shorter T_2_* (Table 4). Fig. 4(b inset) shows the AREX spectra, further suggesting that there no significant difference in the NOE and CEST peaks between the oxygenated and deoxygenated spectra.

**Table 4:** The peak widths and position for APT, amines, NOEs, DS and MT from the LD model fitting.

	APT	Amines	NOE_-1.7ppm_	NOE_-3.5ppm_	DS	MT
Fitted **peak position** averaged over all samples (ppm)	3.55 ± 0.04	2.08 ± 0.02	Fixed at −1.7 ppm	−3.49 ± 0.01	0.02 ± 0.02	0.43 ± 0.19
Fitted **peak width** for oxygenated samples (ppm)	3.5 ± 0.3	1.90 ± 0.09	0.52 ± 0.05	4.7 ± 0.2	1.62 ± 0.04	36.8 ± 4.2
Fitted **peak width** for deoxygenate-d samples (ppm)	3.2 ± 0.1	2.90 ± 0.02	1.3 ± 0.2	4.4 ± 0.2	2.17 ± 0.07	40.0 ± 1.9

Fig. 4(b) and (c) suggest considerable changes in the z-spectra after lysing the blood, highlighted in Fig. 4(d) which directly compares z-spectra from fresh and lysed cRBCs. Whilst the most significant changes in the spectra are due to the changes in relaxation times, the NOE_-1.7ppm_ signal is not apparent in the lysed blood despite the reduction in DS in this region of the spectrum (Fig. 4(d) inset). Fig. 4(e) shows there is little change in the z-spectra on clotting the concentrated RBCs except for a reduction in the NOE_-1.7ppm_ signal (Fig. 4(e) inset).

The LD model fitted the spectra well and the results are shown in Table 4. In the LD fitting we assumed a symmetric MT lineshape as observed in the *ex vivo* blood and sagittal sinus spectra. However, we also attempted to fit an asymmetric MT lineshape but the AREX results were unchanged.

**Fig. 5:**
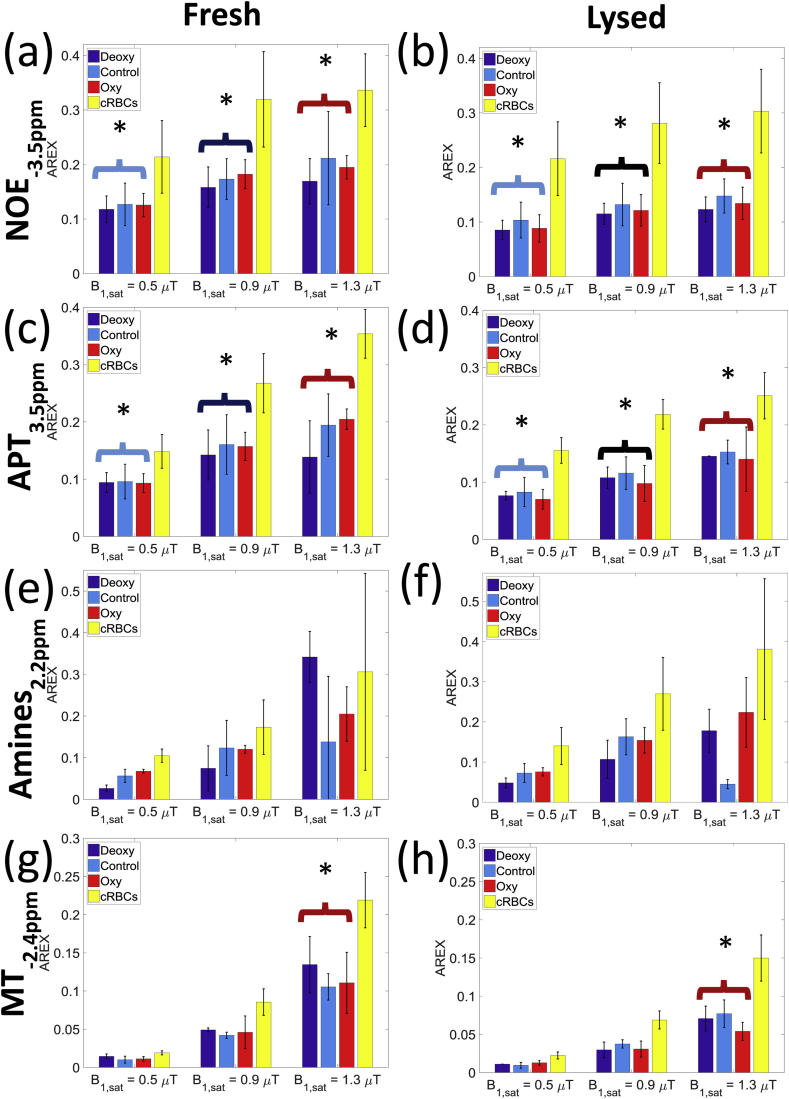
The calculated AREX from the Lorentzian lineshape fitting, showing the NOE_-3.5ppm_, APT_3.5ppm_ and Amine_2.2ppm_ and MT_-2.4ppm_ signals for the deoxygenated, control, oxygenated and cRBCs blood samples for the target saturation powers 0.5, 0.9 and 1.3 μT. Statistical (2 way ANOVA) tests showed that were significant differences between the fresh and lysed blood samples, across the saturation powers.

Fig. 5 shows the AREX signals for the NOE_-3.5ppm_, APT, amine and MT for both the fresh and lysed oxygenated, deoxygenated, control and concentrated RBCs blood samples, for the three saturation powers separately. NOE_-1.7 ppm_ AREX values could not be calculated as they tend to infinity due to their close proximity to the DS peak (Heo et al., 2017). The NOE_-3.5ppm_, APT, and amine AREX signals showed an average 40% increase between the control sample and concentrated RBCs samples.

The NOE_-3.5ppm_ AREX signal showed a trend to be higher at low B_1_ power for all samples, whereas the APT, amine and MT pools sizes tended to increase across all B_1_ powers, except for the control sample amine AREX signal which dropped at high power, possibly because of its close proximity to the DS peak.

Comparing the control, oxygenated and deoxygenated blood samples for each power with 2 way-ANOVA, the NOE_-3.5ppm_ AREX pool showed no significant change with oxygenation (p = 0.59), but upon lysing fell by an average of 27% for all samples (p = 0.003). The APT signal also showed no significant change on oxygenation (p = 0.67) and fell by an average of 22% (p = 0.01) on lysing. There was no significant difference between the fresh and lysed amines (p = 0.06). The MT signal fell by an average of 60% on lysing (p = 0.017) for the 1.3 μT saturation (the low power signal was too small to compare).

**Fig. 6:**
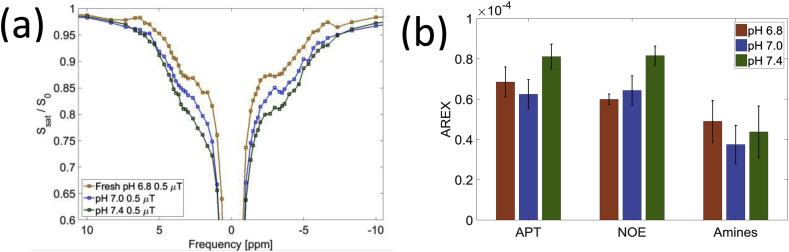
(a) Z-spectra for blood with pH 6.8, 7.0 and 7.4 showing an effect on the APT and NOE_-3.5ppm_ signal change at B_1_ corrected target saturation power of 0.5 μT (b) AREX for the APT, NOE_-3.5ppm_ and Amines at a target power of 0.5uT respectively for the blood with pH 6.8, 7.0 and 7.4.

Fig. 6 shows effects of pH on the z-spectra and AREX signal when the blood was buffered to pH 6.8, 7.0 and 7.4, and suggests that both the NOE_-3.5ppm_ and APT exchanges are base catalysed.

**Fig. 7:**
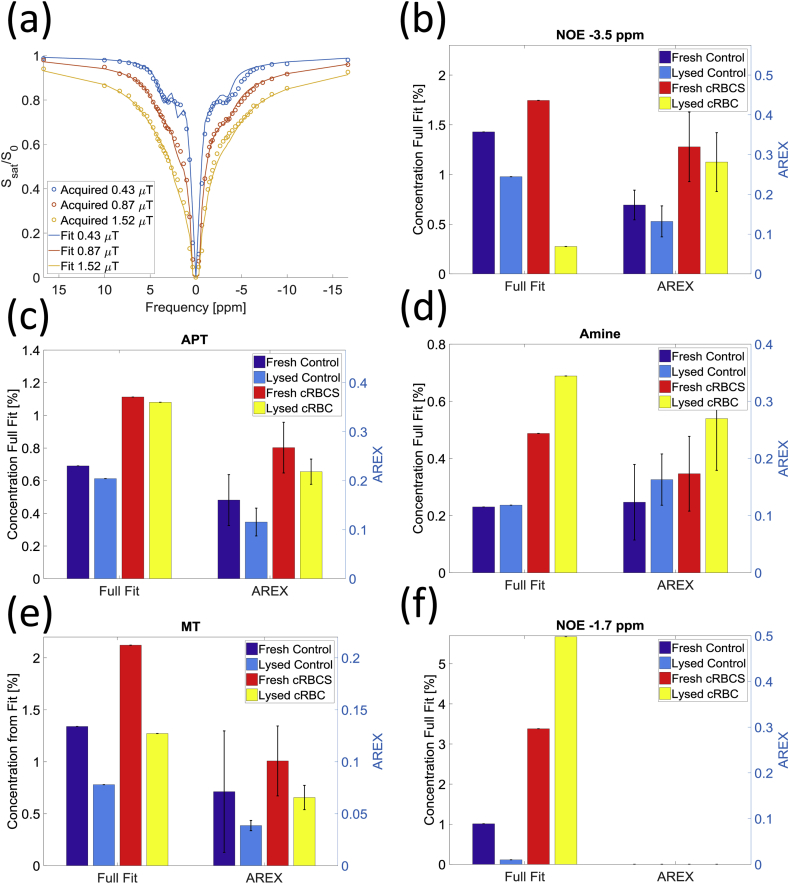
(a) 16 parameters fitted using the 6 pool Bloch McConnell equations on the control fresh blood sample. The fit results are presented in Table 5 (b–f) Estimated pool size of NOE_-3.5ppm,_ APT, Amines MT and NOE_-1.7ppm_ from the final 5 parameter Bloch McConnell fit. vs. the AREX results for the target B_1_ saturation of 0.9 μT are also shown. The AREX signal cannot be calculate for NOE_-1.7ppm._

Fig. 7(a) shows the Bloch-McConnell 6-pool model fitted to z-spectra obtained for the fresh and lysed control blood samples as well as for the fresh and lysed concentrated RBCs for three different combination of B_1_ saturation powers. Table 5 shows the results of the 16 parameter fit with ranges of fitted values consistent with halving the SNR as an indication of the uncertainty in the fit. The apparent range T_2_s of the APT and amine exchanging proton pools in Table 5 are shorter than those previously found in grey matter, whilst the T_2_ of the MT is longer.

**Table 5:** Results of the 16 parameter fit to the 6 pool Bloch-McConnell fitting of the z-spectra acquired on the control blood samples, together with an indication of the uncertainty in the fit.

	APT	Amines	NOE _-1.7ppm_	NOE_-3.5ppm_	MT
Pool concentration (%)	0.78	0.39	1.80	1.44	1.05
	0.78–1.29	0.01–0.64	0.58–2.80	0.44–2.47	0.36–1.30
Exchange rate (Hz)	33.1	338.7	6.35	4.9	10.3
	6.3–50.0	120–481	5.3–15.6	2.9–10	10.3–27.8
T_2_ (ms)	1.4	30.7	1.5	5.9	0.093
	1.4–6.5	13.6–58.7	1.5–8.4	1.4–9.0	0.023–0.093

Fig. 7(b–f) show the results of the final 5 parameter fit to the 6 pool model for the fresh and lysed control and concentrated samples and compares the results to AREX for the mid saturation power (taken from Fig. 5).

## Discussion

Significant CEST signals can be detected from major blood vessels on *in vivo* MRI and here we have shown that these signals can also be detected in blood *ex vivo*. Amide and amine (which are related to proteins and amino acids) signals and two NOE peaks were detected in the z-spectrum from blood. The results indicate that these signals originate largely from RBCs and do not depend significantly upon blood oxygenation. Upon lysing the blood we observed a significant decrease in the amide, NOE_-3.5ppm_ and MT AREX signal and the NOE_-1.7ppm_ peak disappeared (inset in Fig. 4(d)). The source of NOE_-3.5ppm_ signal is currently under debate but is assumed to derive from aliphatic and olefinic protons in mobile proteins involving direct dipole-dipole interactions and exchanged relayed interactions (Jones et al., 2013) (in the brain NOE_-3.5ppm_ has been reported to be closely related to myelin (Mougin et al., 2013)).

Any z-spectrum signal from blood is crucial for the interpretation of endogenous z-spectrum signals detected *in vivo*. For example the effect of a change in blood volume must be considered when studying a change in APT signal from a tumour, or the effects of possible blood break down products must be considered when studying a patient with a stroke.

Fig. 4(a) indicates that the z-spectrum features arise largely from the red blood cells rather than the plasma. Signals were observed in the z-spectrum of plasma (Fig. 4(a)) probably related to amines in albumin and other plasma proteins and possibly residual red blood cells, but the longer T_1_ of the plasma (Fig. 2(b)) will have accentuated the exchanging peaks. To account for this we calculated the T_1_ corrected AREX spectra (Fig. 4(a) inset) and found that the NOE, APT and MT signals were more than 8 times greater in the cRBC samples than the plasma samples.

There was an increase in all z-spectrum features in the concentrated red blood cell sample (Fig. 4(a) and (b)), and the AREX which gives a relative signal for NOE_-3.5ppm_ and APT, showed there was an average 40% increase between the control, oxygenated or deoxygenated samples and, the concentrated RBCs samples, which corresponds to the measured increase in haematocrit (42%). Similar results were obtained for the BM fit except for NOE_-3.5ppm_ which was underestimated for lysed cRBCs. The pH was measured before and after scanning, and a drop in pH of 0.2–0.3 for all the blood samples was detected which would have modified the results since exchange rates of amides (and amines to a lesser extent) are base catalysed (Zhou et al., 2003).

Deoxygenated haemoglobin is highly paramagnetic and oxygenated haemoglobin is slightly diamagnetic relative to plasma, whilst dissolved oxygen makes plasma paramagnetic. In deoxygenated blood this results in magnetic field gradients around the red blood cells leading to the observed shorter T_2_* and broader DS peak (Weisskoff and Kiihne, 1992). The net frequency of the sample will also have been shifted by 0.48 ppm up-field (Gasparovic and Matwiyoff, 1992), but we only considered relative frequency shifts. A small negative variation of T_1_ with oxygenation might have been expected (d'Othée et al., 2003, Blockley et al., 2008) but in fact the control blood had the longest T_1_. This was probably because the oxygenated samples all had an SO_2_ of 100% causing oxygen to be dissolved in the plasma reducing T_1_ (Ma et al., 2016). The T_1_ of (not oxygenated) plasma was similar to CSF. The main observable difference between the z-spectra of oxygenated and deoxygenated blood (Fig. 4(b)) was the narrower DS peak consistent with the fact that the samples had similar T_1_s and very different T_2_*s. However, the results of the AREX fitting, was performed separately at each power, do suggest some trends for the amine signal to increase with oxygenation, possibly related to the change in conformation of the haemoglobin molecule (which contains histadine) on oxidation (the results at high power are less reliable due to the effects of DS on the AREX calculation close to resonance).

After lysing, T_2_* increased for all blood samples, which is expected because of the homogenization of the sample. T_1_ decreased particularly for the deoxygenated sample which may be related to the fact that lysing can lead to the formation of methaemoglobin which has a particularly high T_1_ relaxivity. After lysing the DS peak was narrowed and the MT baseline, CEST and NOE features reduced in amplitude which would be consistent with the known effects assuming an increase in T_2_* and a reduction in the T_1_. Both AREX and the BM analysis attempt to correct for the effects of such changes in T_1_, and indicated a significant reduction in NOE_-3.5ppm_, APT and MT on lysing. The reduction in MT, APT and NOE_-3.5ppm_ upon lysing suggests that these are related to the semi-solid intracellular and membrane components which are denatured as the RBCs are destroyed. The AREX NOE_-3.5ppm_ in the plasma (Fig. 4(a)) is likely to be associated with albumin but was measured to be 7 times less that the AREX NOE_-3.5ppm_ in the lysed cRBCs (results not shown). Lysing is associated with both conversion of haemoglobin to methaemoglobin and changes in the cell membrane, but the trend for the NOE_-3.5ppm_ AREX signal to be reduced particularly for the oxygenated blood, might point to this effect originating in haemoglobin.

Clotting had surprisingly little effect on the z-spectrum. However both lysing and clotting caused the NOE_-1.7ppm_ peak to disappear. This could not be quantified by AREX since the NOE_-1.7ppm_ peak was so close to the DS peak. Similarly the BM fit did not properly fit the NOE_-1.7ppm_ for the cRBCs, probably due to its sensitivity to the estimate of T_2_. AREX quantification of amines has only been successfully employed at 9.4T (Zhang et al., 2017b). Examining all the z-spectra the NOE_-1.7 ppm_ signal is visible in the unclotted, unlysed blood, and particularly in cRBCs, and at low pH. This could be related to a result recently reported by *Zhang* et al. who examined an 'NOE mediated MT effect around −1.6 ppm’ and observed a strong contrast difference between healthy and stoke tissue in rats (Zhang et al., 2016). Future work will focus on determining this frequency more precisely. At increased ionic strength (or osmolarity) red blood cells can form echinocytes (or burr cells) losing their discoid shape and gaining small protrusions on their surface, associated with separation of the lipid bilayer from the underlying membrane skeleton (Liu et al., 1989), and similar effects are observed *in vivo* in various situations where the cells are under stress. Previous work using ^1^H NMR suggests that echinocyte formation is associated with changes in the distribution of phospholipids between the two leaflets of the cell membrane (Pages et al., 2010). It has recently been shown that reconstituted egg phospholipids show an NOE_-1.7ppm_ peak (Zhang et al., 2017a). It seems possible that morphological transformation of the cell membrane is in some way responsible for the change in the NOE_-1.7 ppm_ peak although further work including optical microscopy is required to confirm this.

The increase in the amine signal estimated by AREX with B_1_ is consistent with amines being in faster exchange than the other pools and thus requiring more B_1_ saturation power to observe magnetisation exchange of the protons of interest. The discrepancy for the control sample at high power is due to the broad DS peak which perturbed the LD model fit for that sample. There is good agreement between AREX at medium power and the BM model as shown in Fig. 7.

In this work the data were analysed quantitatively in two ways: by fitting to an LD model with AREX analysis which yields a relative CEST or NOE proton signal but does not measure the pool sizes, and by fitting to a BM model which gives absolute proton pool sizes (relative to the water pool) and an estimate of T_i2_, and k_i,ex_ for the exchanging pools. Fig. 7 shows generally good agreement between the relative pool sizes estimated from the two methods. However only relative quantification is possible with AREX. For instance for LD fitting, spline interpolation was used to correct for B_1_ field inhomogeneities at 7T (Windschuh et al., 2015, Jones et al., 2011), but the AREX results clearly varied with B_1_ saturation power in a way that depended on exchange rate (Jones et al., 2011). AREX is also unable to cope with coalescing peaks, particularly close to the water peak. Furthermore the AREX theory was derived for a spin system in a steady state (Jones et al., 2011), which may not be achieved for samples with longer T_1_ using a finite train of saturation pulses. Simulations of the presaturation pulse trains used in our experiments (40 pulses in 2.4 s) showed the system achieved approximately 95% of steady state at the end of the pulse train. The full BM fit does not require the system to reach steady state. The BM fit was performed on a subset of the samples which allowed us to test whether it was possible to detect changes expected due to changes in concentration, but it was still compromised for NOE_-1.7ppm_ probably due to errors in the estimate of T_2_ which are likely to have also affected the fit to the amine peak. The exchange rates and relaxation times measured in the control blood sample from the BM fit were generally of the same order of magnitude as the values measured in the brain (van Zijl et al., 2017). The T_2_ values were somewhat shorter than previously reported, despite the fact that the blood was less structured than brain tissue. This may indicate that the peaks identified actually correspond to multiple peaks which have not been accounted for in the BM model. The exchange rates measured for amines suggest that they may originate more in guanidinium (which can be found in creatine and the arginine side groups in proteins) at 2 ppm rather than glutamate at 3 ppm (van Zijl et al., 2017) since the guanidinium proton group has a lower exchange rate than amine protons (Chen et al., 2017). In general the relaxation times measured were shorter which might be expected given the reduced structure in blood compared to grey matter. These values can provide starting values for future investigations of blood and possible multicompartmental modelling of tissue. Future work will use continuous wave saturation to provide improved spectra and will fit a range of samples to explore the effects of blood oxygenation and pH on T_i2_, and k_i,ex_.

## Conclusion

This work has shown that the CEST and NOE effects seen in z-spectra of *ex vivo* blood originate primarily from the RBCs and that the z-spectrum from blood shows a diverse range of CEST and NOE effects, some of which depend on whether or not the cells have been lysed. These results are crucial to our interpretation of *in vivo* z-spectra in pathology, where changes in blood volume need to be considered.
